# Influence of Lowering the pH Value on the Generation of Fibrous Structures of Protein Gels with Different Network Types

**DOI:** 10.3390/gels10030173

**Published:** 2024-02-29

**Authors:** Felix Ellwanger, Melanie Fuhrmann, Heike P. Karbstein, Gabriela Itziar Saavedra Isusi

**Affiliations:** 1 Karlsruhe Institute of Technology (KIT), Institute of Process Engineering in Life Sciences, Food Process Engineering (LVT), Gotthard-Franz-Straße 3, 76131 Karlsruhe, Germany; 2 Thermo Fisher Scientific, Pfannkuchstr. 10–12, 76185 Karlsruhe, Germany

**Keywords:** extrusion, meat substitute, gel formation, gel strength, structuring mechanism

## Abstract

High-moisture extrusion of plant proteins to create meat-like structures is a process that has met with increasing attention in the recent past. In the process, the proteins are thermomechanically stressed in the screw section of the extruder, and the resulting protein gel is structured in the attached cooling die. Various protein sources, notably soy protein isolate (SPI) and wheat gluten, are used to form gels with different networks: SPI creates a physical, non-covalent network, while gluten forms a chemical, covalent one. The food industry frequently adds weak acids to modify taste and shelf life. However, it is known that a change in pH affects the gelation behavior of proteins because the repulsive forces within and between the proteins change. The research reported here was carried out to investigate for the two proteins mentioned the influence of pH modification by the addition of citric acid and acetic acid on gel formation and the meat-like structures produced. For this purpose, materials and parameters were screened using a closed cavity rheometer, followed by extrusion trials at pH 7.36–4.14 for SPI and pH 5.83–3.37 for gluten. The resulting extrudates were analyzed optically and mechanically, and protein solubility was tested in a reducing buffer. For both protein systems, the addition of acid results in less pronounced meat-like structures. At decreasing pH, the complex viscosity of SPI increases (from 11,970 Pa·s to 40,480 Pa·s at 100 °C), the generated gel becomes stronger (strain decreased from 0.62 to 0.48 at 4.5 × 10^5^ Pa), and the cross-linking density grows. For gluten, a decreasing pH results in altered reaction kinetics, a more deformable resulting gel (strain increased from 0.7 to 0.95 at 4.5 × 10^5^ Pa), and a decreased cross-linking density. Solubility tests show that no additional covalent bonds are formed with SPI. With gluten, however, the polymerization reaction is inhibited, and fewer disulfide bonds are formed.

## 1. Introduction

In recent years, meat substitutes have become increasingly popular. This may be due to consumers’ increasing awareness of the ethical, environmental, and health impacts of meat consumption [1,2]. As plant-based protein sources, meat substitutes are supposed to represent a sustainable and healthy alternative to animal meat [3]. High-moisture extrusion is a common process for the production of such meat substitutes. In the high-moisture extrusion process, proteins are mixed with water and other ingredients, thermomechanically stressed in the screw section, and then pressed through a cooling die [4,5]. In the cooling die, anisotropic structures are generated that are supposed to imitate muscle meat [6]. Various hypotheses have been proposed regarding the origin of these anisotropic, meat-like structures, with two being most widely accepted. Firstly, it is argued that proteins above the denaturation temperature align and rearrange within the shear flow in the cooling die. Upon cooling, the realigned protein gel solidifies [7]. The second hypothesis assumes the formation of a multi-phase system, in which phase separation and phase deformation occur [8]. Wittek et al. showed for soy protein isolate that a water-rich, low-protein phase and a protein-rich, low-water phase are formed, which are aligned in the cooling die [9,10]. It must be taken into account, however, that the different processes may take place simultaneously and influence each other. Protein denaturation and rearrangement may induce phase separation and thus support the formation of a multi-phase system. A clear overview of the different hypotheses as well as new considerations can be found in the recent review by van der Sman and van der Goot [11].

In protein-rich food gels, such as meat substitutes, yogurt, and cheese, the protein gel formed by gelation plays a decisive role in the product’s texture. The gels that form can either be physical, i.e., formed by non-covalent bonds (e.g., hydrophobic, ionic), or chemical, i.e., additionally reinforced by covalent bonds (e.g., disulfide bonds) [12]. Regardless of the type of gel, the bonds between the molecules are referred to as cross-links. The gelation behavior and, hence, cross-linking density depend on both intrinsic factors that are related to the protein (i.e., amino acid composition, molecular weight) and extrinsic factors that are related to the conditions surrounding the proteins (i.e., pH, ionic strength, temperature) [13]. While the influence of many intrinsic and extrinsic factors is extensively studied for meat substitutes, the influence of pH modification has not yet been investigated in sufficient detail.

Recently, two papers were published on pH modification in the high-moisture extrusion of pea protein isolate and concentrate, wheat gluten, and rice protein isolate [14] and low-moisture extrusion of pea protein isolate [15]. These outstanding publications reveal differences in the micro- and macrostructures of the produced extrudates. The gelation process and differences in the present gel structure are not discussed in detail.

However, the influence of pH modification is studied well in many low-concentration protein solutions and protein-stabilized emulsions, such as yogurt and cheese. In this context, the process of acid-induced gel formation is referred to as acidification. The principle of gel formation is based on the effect whereby, when lowering the pH in the direction of the protein isoelectric point, the surface charge of the proteins decreases, repulsion no longer occurs, and aggregation up to gel formation takes place [13,16].

In addition to the altered gel formation mechanisms, a change in the pH value is also expected to alter the solubility of the proteins [17]. For soy protein, for example, it is known that solubility is lowest at pH values of 4–5 and highest at a pH value of 2 [18]. For a change in the pH value of gluten, it is known that the solubility is low at a neutral pH value but is fair at a pH of 4 or lower [19]. Although meat substitutes are highly concentrated systems that are not a solution as such, it can be assumed that protein solubility has an influence on the structuring of meat substitutes. In particular, it could have an influence on the formation of the multiphase system. However, this has not yet been investigated in detail and is also beyond the scope of this study.

In this work, the influence of pH modification on the gel forming and structuring of meat substitutes was investigated using two common proteins forming gels with different networks, soy protein isolate (SPI) and vital wheat gluten (referred to as gluten). SPI predominantly forms non-covalent bonds and therefore is considered a physical gel [10,20,21]. The isoelectric point (IEP) of SPI ranges between pH 4 and 5 [22,23,24]. Gluten proteins are additionally cross-linked by covalent disulfide bonds and represent a chemical gel [25,26,27,28]. The IEP of gluten ranges between pH 7 and 8 [29].

The research question of this study is how the change in pH influences the gelation and thus the formation of meat-like structures in high-moisture extrusion process of meat substitutes. On the basis of the two protein systems, the following hypotheses are made:

The formation of a physical SPI gel is strengthened by a change in pH towards the IEP due to a decrease in electrostatic repulsive forces, resulting in an increased cross-linking density. To deform a stronger gel necessary to create meat-like structures, higher strain and shear stresses are required [9]. Accordingly, the structure is expected to be less pronounced under constant extrusion conditions.

For a chemical gluten gel, a change in pH away from the IEP results in an increase in electrostatic repulsive forces between proteins. This may lead to fewer physical cross-links, and polymerization into a chemical gel is inhibited. A weak, unstable protein gel may result. However, the weaker gel can be better deformed in the die, although it may be doubted whether there are sufficient cross-links for a stable structure in the absence of covalent bonds.

In order to address the research question and hypotheses, first, temperature ramps were performed in a closed cavity rheometer to determine changes in gelation behavior and gel strength. Furthermore, extrusion tests were carried out on a laboratory scale. The resulting extrudates were observed optically and investigated by classical mechanical methods to obtain further information about the resulting gels. Finally, solubility tests in reducing buffer were carried out to justify the differences in the resulting gels.

## 2. Results and Discussion

### 2.1. Material Screening Based on Measurements in the Closed Cavity Rheometer

**Figure 1:**
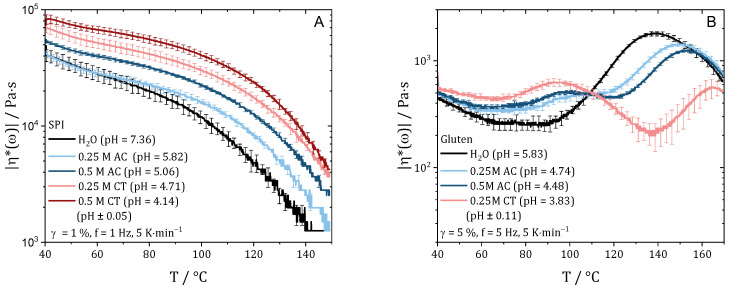
Temperature ramp of SPI (**A**) and gluten (**B**) doughs prepared with water and acids in different concentrations.

Temperature ramps were performed to obtain information about the gel structure and to predict changes in the reaction and gelation behavior. Figure 1 shows the magnitude of the complex viscosity (referred to as viscosity) plotted over the temperature for SPI (A) and gluten (B) doughs prepared with water and acids. For protein doughs, a decrease in viscosity can be related either to an increase in molecular mobility or, at elevated temperatures, to degradation reactions. An increase in viscosity can be attributed to aggregation or polymerization reactions [26,30,31].

For all SPI samples (Figure 1A), viscosity is observed to decrease with the increase in temperature for the whole temperature range. As described before, the decrease in viscosity is due to the higher mobility of the molecules. The absence of an increase in viscosity indicates that no polymerization takes place, as already described in the literature [32].

The addition of the acid causes a horizontal shift of the viscosity curves. The viscosity increases at the starting temperature (40 °C), indicating that the proteins are pre-coagulated. The value of the viscosity at a temperature of 100 °C (taken from Figure 1A) increases with the decrease in pH value, ranging from 11,970 ± 1490 Pa·s for pH 7.36 to 15,970 ± 1000 Pa·s for pH 5.82, to 22,350 ± 719 Pa·s for pH 5.06, to 30,090 ± 2520 Pa·s for pH 4.71, to 40,480 ± 2610 Pa·s for pH 4.14. As assumed in the hypothesis, the increasing viscosity with the decrease in pH shows that the cross-linking density increases. This can be attributed to the pH moving towards the IEP of SPI, which reduces repulsion of the molecules.

The addition of acid to gluten leads to a shift away from the IEP. Even if repulsion between the molecules increases, a cross-linking of the proteins can be detected for all investigated samples. The viscosity of the dough with 0.5 M citric acid was too low to be measured with an acceptable signal-to-noise ratio and, hence, is not shown.

For the gluten/water mixture with a pH of 5.83, the typical behavior of gluten is obtained. There is an initial decrease in viscosity up to a temperature of about 60 °C due to the increasing mobility of the molecules with the increase in temperature. As the temperature is further increases, the slope changes (reaction onset), and a large increase in viscosity is observed from a temperature of about 90 °C. This increase can be linked to the polymerization reaction of the glutenin and gliadin fractions of gluten [26]. The maximum is reached at 138 °C, after which the viscosity decreases again due to the increasing mobility and/or degradation reactions.

Contrary to SPI, no horizontal shift in viscosity is observed for gluten due to the modification of the pH by adding acids. At low temperatures (<70 °C), viscosity is slightly increased with the decrease in pH. Interestingly, polymerization appears to start at lower temperatures with the decrease in pH. The increase in viscosity starts at a temperature around 90 °C for pH 5.83, 83 °C for pH 4.74, 80 °C for pH 4.48, and 80 °C for pH 3.83. For the pH-modified samples, there is a plateau in viscosity at a temperature of 102 °C for pH 4.7 and 98 °C for pH 4.48 and even a decrease in viscosity at a temperature of 92 °C for pH 3.83. However, a second increase in viscosity can be observed at temperatures of 115 °C for pH 4.7, 123 °C for pH 4.48, and 138 °C for pH 3.83. After reaching viscosity maxima at temperatures of 149 °C for pH 4.8, 152 °C for pH 4.48, and 165 °C for pH 3.83, it seems like the viscosity curves coincide again. The reason for the different viscosity curves, especially those with two regions of viscosity increase, is not clear.

However, when the pH is modified by the addition of acids, the ionic strength of the solution is also changed. It is known that the structure of protein networks can also be changed by changing the ionic strength [13]. Accordingly, NaCl solutions were prepared with the ionic strength of the strongest acid (0.5 M citric acid), and temperature ramps were performed with the CCR. No significant change was observed (see Appendix A), which is why the changes are attributed to the shift in pH exclusively.

Based on the changed gel strength for SPI and the changed polymerization behavior of gluten, we assume that the addition of acid affects the structuring process in high-moisture extrusion. The viscosity of the SPI doughs increases with the decrease in pH, which will have an influence on the flow profile in the cooling die. From these data, we expect that the meat-like structures are not as pronounced because higher shear rates and higher strain rates are required to deform the gel, and the effects of plug flow and wall slip may be enhanced. For gluten, no clear expectation can be stated. On the one hand, it should be easier to deform the weaker gels due to the decreased viscosity at moderate temperatures (110–140 °C). On the other hand, lack of covalent cross-linking due to a change in polymerization rate can result in a gel that is too deformable and weak.

### 2.2. Changes in the Macroscopic Structure of Meat Substitutes Due to the Addition of Acids

**Figure 2:**
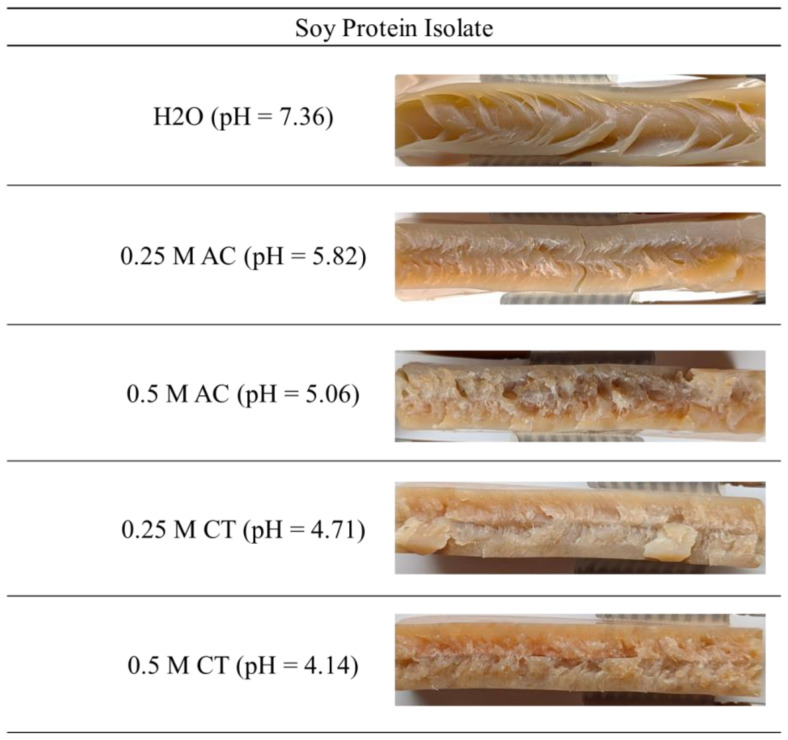
Soy protein isolate extrudates produced with water, 0.25 M acetic acid, 0.5 M acetic acid, 0.25 M citric acid, and 0.5 M citric acid.

**Figure 3:**
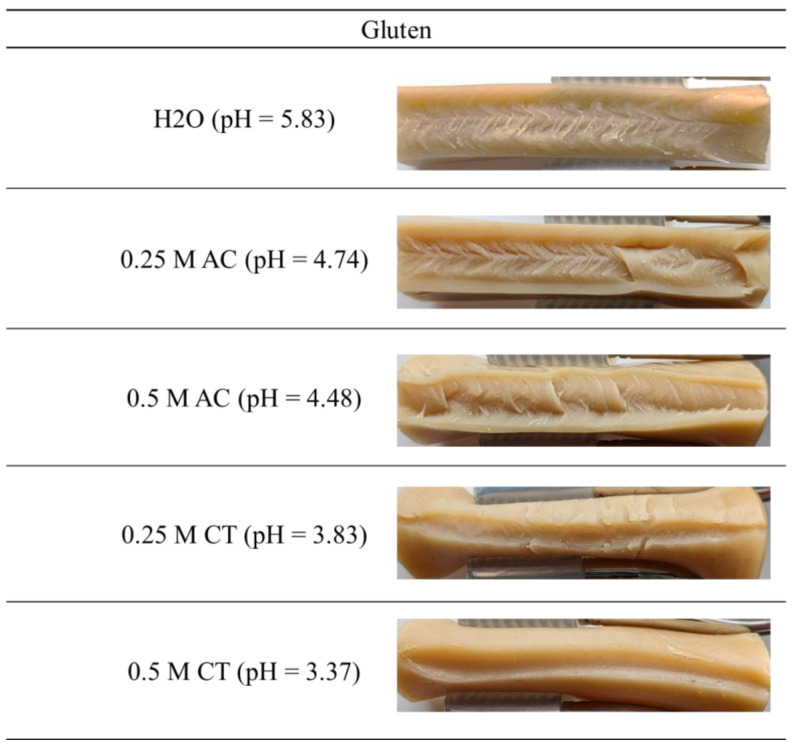
Gluten extrudates produced with water, 0.25 M acetic acid, 0.5 M acetic acid, 0.25 M citric acid, and 0.5 M citric acid.

For the SPI (Figure 2) and gluten extrudates (Figure 3) prepared with water, anisotropic fibrous structures can be produced, as already shown in the literature [9,33]. Parabolic flow profiles are obtained for both protein types, with SPI exhibiting larger, longer fibers than gluten. As expected from the CCR experiments, both proteins exhibit a change in structure after extrusion with acids.

The fibrous structure of the SPI samples is less pronounced at pH 5.82. As the pH decreases further, no fibrous structure can be seen at all. The samples become very brittle and solid. The rheological data obtained with the CCR indicate that the viscosity increases with the decrease in pH. As no fibrous structure is visible after extrusion and no parabolic flow profile can be seen, it is hypothesized that the flow profile changes. It is possible that the shear stress is no longer sufficient to make the material flow, and a kind of plug flow prevails in the die. Additionally, the multi-phase system and phase separation may be changed due to the addition of the acid as a stronger gel is formed and the formation of a second phase could be suppressed. The change in the solubility of the proteins could also have an influence on the formation of the multiphase system, as it has been shown in the literature that a water-poor, protein-rich phase and a water-rich, protein-poor phase occur [10]. Cryo-imaging or Micro-CT imaging may provide some more information in the future [10]. It would also be interesting to see whether it is possible to create fibrous structures by changing the viscosity of the pH-modified samples, e.g., by increasing the temperature or decreasing the protein concentration.

For the gluten samples, fibrous structures are visible in the samples up to a pH value of 4.48, although they are less pronounced. For the samples with pH 3.83 and 3.37, a fibrous structure is no longer visible. With a decreasing pH and, hence, an increasing distance from the IEP, the extrudates become more elastic and dough-like. This can be attributed to the increased repulsion between the protein molecules as well as to the changed polymerization kinetics displayed by the rheological data obtained with the CCR, which could lead to fewer cross-links. For gluten dispersions prepared in a pH range from 7 to 2.5 and heated at 133 °C for 15 min, Langstraat et al. also reported that the cross-linking reaction was impaired significantly when the pH was lowered [34].

A comparison with high-moisture-extruded meat substitutes with altered pH value from the work of Nisov et al. is only possible to a limited extent, as the anisotropic structure was not visualized. However, the results describing that structuring at lower pH of rice protein, vital wheat gluten, and pea protein concentrate and isolate extrudates requires a higher temperature suggest that the network has been altered by the pH change, and structuring is more difficult to achieve [14].

### 2.3. Influence of the Modification of pH on the Mechanical Properties of Meat Substitute Extrudates

Mechanical analyses provide information about the gel structure, as the cross-linking density correlates with mechanical properties. For this reason, mechanical properties of the extrudate are used to confirm the explanations of the visual observations and CCR measurements.

**Figure 4:**
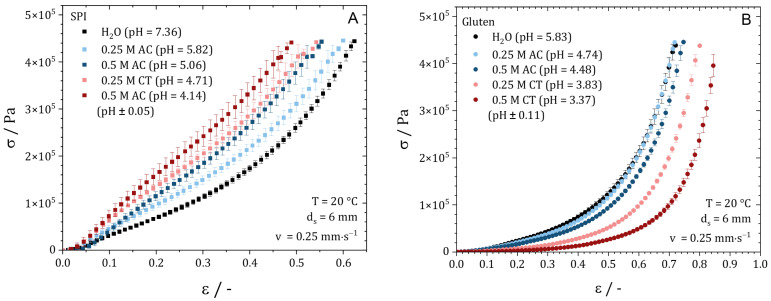
Compression tests of (**A**) soy protein isolate extrudates and (**B**) gluten extrudates produced with water, 0.25 M acetic acid, 0.5 M acetic acid, 0.25 M citric acid, and 0.5 M citric acid.

Figure 4 shows the stress–strain curves for the SPI (A) and gluten extrudates (B). The stress–strain curves for SPI (Figure 4A) confirm the optical description of the extrudate. As the pH decreases and the IEP is approached, the extrudates become stiffer and reach the maximum force at a lower strain. With the decrease in pH, the slope of the curves increases, and a higher force is needed for the same strain. While the extrudates produced with water can still be compressed by up to 62% at maximum force, the extrudate at pH 4.14 can only be compressed by 48% at the same force. Similar observations were reported in the literature for soy protein networks with lower concentrations. Shen et al. reported for a soy yoghurt that the hardness of the yoghurt increases with the decrease in pH [35]. For heat-induced SPI gels, Renkema found that stronger networks were formed closer to the IEP due to the increased attractive interaction energy [20]. The results obtained here and the results presented in the literature indicate that the cross-link density increases with the decrease in pH, as was assumed. Accordingly, the gel becomes stronger, which correlates to the rheological data obtained with the CCR.

Visual inspection of the gluten doughs revealed that the extrudates became more deformable and dough-like with the decrease in pH. These observations are confirmed by the stress–strain curves. With the decrease in pH, the plastic behavior of the gluten extrudates increases, i.e., less force is required for the same deformation, and the sample can be deformed further before the yield point occurs. While the extrudate prepared with water was compressed by 70% at maximum force, a strain of 85% was achieved for the sample with a pH of 3.37. This could be due to the fact that repulsion increases with the decrease in pH and polymerization is inhibited, which results in a lower cross-linking density. This has already been reported in the literature for gluten dispersions [34].

**Figure 5:**
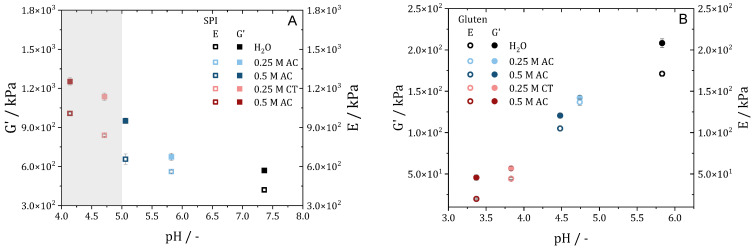
Young’s moduli and storage moduli of soy protein isolate extrudates (**A**) and gluten extrudates (**B**) produced with water, 0.25 M acetic acid, 0.5 M acetic acid, 0.25 M citric acid, and 0.5 M citric acid.

**Figure A2:**
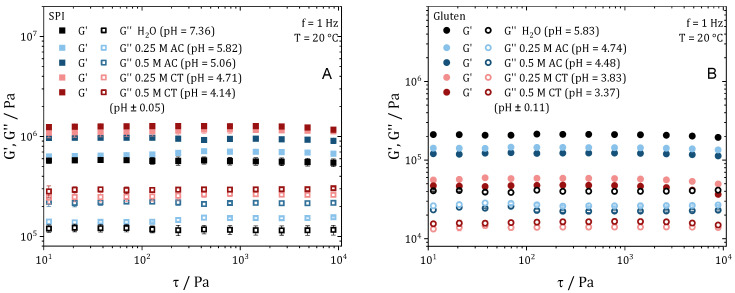
Amplitude tests of (**A**) soy protein isolate extrudates and (**B**) gluten extrudates produced with water, 0.25 M acetic acid, 0.5 M acetic acid, 0.25 M citric acid, and 0.5 M citric acid.

**Figure A3:**
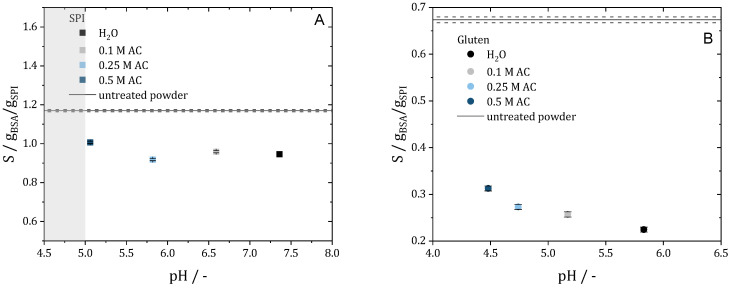
Solubility tests in reducing buffer of (**A**) soy protein isolate extrudates and (**B**) gluten extrudates produced with water, 0.25 M acetic acid, 0.5 M acetic acid, 0.25 M citric acid, and 0.5 M citric acid.

In addition to the compression tests, strain sweeps were conducted on the different extrudate samples. The mean value of the storage modulus in the LVE (10^1^–10^4^ Pa) as well as Young’s modulus were used to estimate the cross-link density [36,37]. Figure 5 shows the storage modulus and Young’s modulus plotted over the pH value. The corresponding strain sweeps are presented in Appendix A Figure A2. For the SPI extrudates (Figure 5A), the storage modulus and Young’s modulus increase with the decrease in pH value. While the absolute values of the storage modulus and Young’s modulus differ, their trends are almost parallel. This indicates that the protein molecules become closer together with the decrease in pH value and more cross-links, and thus a stronger gel is formed, as expected. Solubility tests in reducing buffer (Appendix A Figure A3) suggest that no additional chemical cross-links are formed, as reported in the literature [10]. The additional cross-links formed are due to steric, hydrophobic, ionic, van der Waals interactions or hydrogen bonds. Accordingly, we can confirm our hypothesis that a decrease in the pH in the high-moisture extrusion process leads to SPI forming a stronger gel based on physical interactions, which results in less pronounced meat-like structures.

As already reported, the opposite behavior is found for gluten extrudates. With the decrease in pH, the storage modulus and Young’s modulus decrease with the same tendency. Solubility tests in reducing buffer (Appendix A Figure A3) indicate that with the decrease in pH, fewer disulfide bonds are formed, and the polymerization reaction is inhibited. The decreasing cross-linking density can be explained by two phenomena. On the one hand, repulsion between the protein molecules increases, as the pH moves away from the IEP. On the other hand, the polymerization reaction is inhibited, resulting in fewer covalent bonds. Thus, by lowering the pH during gluten extrusion, we obtain a weaker gel that is easier to deform. However, no anisotropic structures can be generated because there are probably too few cross-links.

## 3. Conclusions

The effects of pH modification on the textural properties of meat analogues were assessed successfully. Soy protein isolate (SPI) and vital wheat gluten were studied as representatives of a physical and chemical gel, respectively.

For gels made from SPI, tests at the closed cavity rheometer and mechanical tests of the meat substitutes produced have shown that the cross-linking density increases with the decrease in pH. Solubility tests showed that the new cross-links must be of a physical nature. The resulting firmer gels made structuring more difficult at a reduced pH value. For the gels produced from gluten, an altered polymerization reaction resulting from a change in pH was observed by screening in the closed cavity rheometer. Mechanical tests of the produced meat substitutes showed that the cross-linking density decreases with the decrease in pH, and solubility tests showed that fewer covalent bonds were formed. Structuring the pH-reduced gels is more difficult, as the gel is not strong enough to hold a structure due to the lack of disulfide bonds. This study shows that the addition of acid for taste or stability can have dramatic consequences in the production of meat substitutes. Accordingly, the process must be redesigned when the recipe is changed.

## 4. Materials and Methods

### 4.1. Materials

Commercial soy protein isolate (SPI) Supro ST from Solae LLC (St. Louis, Mo, USA) and vital wheat gluten from Kröner Stärke (Ibbenbüren, Germany) were used for this study. According to the manufacturers, the protein content of SPI was >90%, and the water content was <6%. The protein content of gluten was >83%, and the water content was <8%. The chemicals for studying protein solubility, sodium chloride, acetic acid, and citric acid, were obtained from Carl Roth GmbH + Co. KG (Karlsruhe, Germany) with a purity > 99.5%.

### 4.2. Dough Preparation for Material Screening with the Closed Cavity Rheometer

Doughs for rheological screening were prepared in a Thermomix from Vorwerk (Wuppertal, Germany). The water content of the protein doughs was adjusted according to the mass flows of the extrusion experiments. Mixtures with moisture contents of 55% (*w*/*w*) for SPI and moisture contents of 45% (*w*/*w*) for gluten were prepared, neglecting the water content of the protein powders. The following liquids were used: tap water; 0.1 M, 0.25 M, and 0.5 M acetic acids; 0.25 M and 0.5 M citric acids; and a sodium chloride solution with a conductivity of 6.96 mS/cm. The doughs were stored for at least 24 h in the refrigerator at 4 °C to ensure equilibrium of hydration.

### 4.3. pH Measurement of the Doughs

The pH of the gluten doughs was measured with a foodcare pH electrode for meat from Hanna Instruments Deutschland GmbH (Vöhringen, Germany). As the SPI doughs were too hard and brittle, the electrode was not suitable for detecting the pH value. Here, a standard method for cereals, flour, and bread was used [38]. First, 5 mL of acetone and 95 mL of freshly boiled and cooled distilled water were mixed. Part of this mixture was placed in a grating bowl with 10 g of SPI dough sample and mixed to form a slurry. The slurry was then transferred without loss with the rest of the acetone/water mixture into a 250 mL Erlenmeyer flask and homogenized. The pH of the mixture was measured with a standard pH electrode while stirring gently with a magnetic stirrer. For verification, the method was also applied to selected gluten doughs and showed a good agreement with the measurements of the foodcare pH electrode for meat.

### 4.4. Material and Parameter Screening with the Closed Cavity Rheometer

Material screening was performed with a rubber process analyzer (RPA) flex, also called a closed cavity rheometer (CCR), from TA Instruments, Inc. (New Castle, DE, USA). This rheometer possesses a closed bi-conical, grooved geometry (radius = 20.625 mm, angle = 7.16°) and is widely used to investigate rheological properties of protein–water mixtures [26,32,39]. The sealed measuring geometry is closed during the measurement with a closing pressure of 4.5 bar, which prevents the evaporation of water at temperatures above 100 °C. The grooves in the geometry prevent the material from slipping.

For material screening, a temperature ramp was performed between 40 °C and 150 °C for SPI samples and between 40 °C and 170 °C for gluten samples. The heating rate was set constant at 5 K/min. Strain sweeps were used to determine the combination of frequency and strain. For SPI samples, a frequency of 1 Hz and a strain of 1% were chosen to ensure a good signal-to-noise ratio within the linear visco-elastic (LVE) region. For gluten samples, a frequency of 5 Hz and a strain of 5% were required to obtain a good signal-to-noise ratio, which was still within the LVE region. Temperature sweeps were carried out in triplicate, and the mean value and the deviation were determined.

### 4.5. Extrusion Process

**Figure 6:**
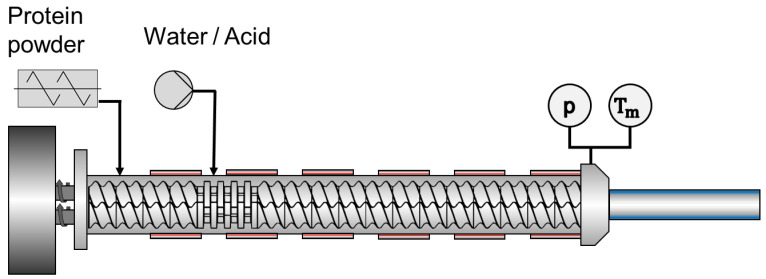
Schematic extruder setup.

Extrusion experiments were performed using a lab-scale twin-screw extruder Process 11 (Thermo Fisher Scientific, Karlsruhe, Germany) mounted with a cooled slit die. The protein powder (SPI or gluten) was dosed via a gravimetrically controlled feeder of Brabender Technology GmbH (Duisburg, Germany). The liquid feed (water, acid, or salt solution) was pumped by a peristaltic pump Masterflex L/S from Cole Parmer (Vernon Hills, IL, USA) into the third barrel element. A schematic drawing of the process is given in Figure 6. The screw had a length to diameter ratio (L/D) of 40. It was mainly made of conveying elements, with a 1.5 L/D 90° kneading block installed after liquid dosing. The screw speed was kept constant at 600 rpm for all extrusion experiments. The dimensions of the cooling die were H × W × L: 4 × 19 × 125 mm, and temperature of the cooling die was set to 30 °C. Mass flow rates of the proteins, mass flow rates of the liquid feeds, and the barrel temperature profile can be found in Table 1. Tap water; 0.1 M, 0.25 M, and 0.5 M acetic acid; and 0.25 M and 0.5 M citric acid were used as liquid feeds. The extrudates obtained were cut into pieces and frozen for storage until further analysis. As shown in the study by Nieuwland et al., it is assumed that the freezing of the extrudates does not affect the meat-like structure and therefore has no influence on the subsequent analysis [40].

### 4.6. Visual Inspection

**Figure 7:**
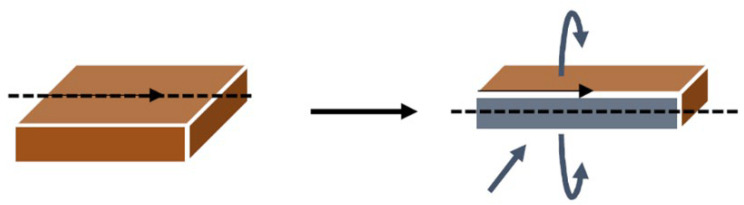
Representation of sample preparation: Thin black arrow—flow direction, dashed line—cutting line, gray plane—photographed plane.

To visualize changes in the anisotropic structure, the extruded samples were thawed overnight and heated to room temperature. Figure 7 shows how the samples were prepared before pictures were taken.

### 4.7. Compression Test

Compressive stresses of the extruded samples were measured with a stress-controlled HAAKE MARS 60 rheometer (Thermo Fisher Scientific, Karlsruhe, Germany). Samples with a round cross-sectional area of 6 mm in diameter were cut out of the center of the extrudates. The upper plate of the rheometer was closed with a velocity of 0.25 mm/s, and a stress–strain diagram was recorded. The measurements were performed up to a maximum force of 45 N. Young’s modulus was calculated from the linear region of the stress–strain diagram using Equation (1) as described by Willats et al. [41] and Fraeye et al. [42], where E is Young’s modulus, F the measured force, A the sample’s cross-sectional area, and ε the strain.

(1) $$E=\frac{F}{A*\varepsilon }$$

Young’s modulus was calculated for a strain of 4–12% for SPI and 10–20% for gluten (linear fit R^2^ > 0.99). Compression tests were carried out in triplicate, and the mean value and the deviation were determined.

### 4.8. Rheological Measurement of the Extrudates

Strain-sweep experiments of the extrudates were performed using a stress-controlled HAAKE MARS 60 rheometer from Thermo Fisher Scientific (Karlsruhe, Germany). A serrated plate geometry was used with a diameter of 20 mm. Samples with a round cross-sectional area of 20 mm in diameter were cut out of the center of the extrudates. The gap width was set to 3.4 mm for the SPI extrudates and 3.0 mm for the gluten extrudates. The measurements were performed at a temperature of 20 °C, at a frequency of 1 Hz, and a stress between 1 and 10^5^ Pa. Before each measurement, all samples were allowed to sit 120 s to rest and equilibrate. Strain-sweep experiments were carried out in triplicate, and the mean value and the deviation were determined.

### 4.9. Protein Solubility

To investigate the formation of covalent bonds during the extrusion process, the solubility in a non-reducing buffer was studied. The buffer was prepared with 0.132 M sodium dihydrogen phosphate, 0.68 M disodium hydrogen phosphate, 0.05 M sodium chloride, 0.0173 M sodium dodecyl sulfate (SDS), and 8 M urea. The extrudates as well as raw protein powders were pre-dried at 50 °C for 24 h, ground, and sieved to a particle size < 280 µm. A total of 10 mg of the sample was mixed with 20 mL buffer, vortexed for 30 s, and dissolved at room temperature for 24 h in a rotary shaker from Edmund Bühler GmbH (Bodelshausen, Germany) at 200 rpm. To obtain the supernatant for determining the protein concentration, a 5920 R centrifuge from Eppendorf AG (Hamburg, Germany) was used at 4310× *g* for 50 min. The absorbance at 280 nm of the supernatant was analyzed in an Evolution 201 spectrophotometer by Thermo Fisher Scientific Inc. (Waltham, MA, USA). A bovine serum albumin (BSA) calibration curve was obtained to calculate relative protein concentrations (concentration range 0.005–2 mg BSA/mL buffer, R^2^ > 0.99). Solubility and absorbance measurements were repeated three times each, resulting in a total of nine absorbance values per sample.

### 4.10. Statistical Analysis

Data of the analyses of the complex viscosity at T = 100 °C for SPI and analyses of the storage modulus of the extrudates was assessed via multifactorial analysis of variance (ANOVA) and a Turkey test as a post hoc test. Dissimilarities in the samples were considered statistically relevant at a level of *p* ≤ 0.05. The software OriginPro 2019 v9.6 (OriginLab Corp., Northampton, MA, USA) was used for the statistical analysis, calculation of averages, and standard deviations.

## Figures and Tables

**Table 1:** Mass flow rates of the protein powder ($\dot{m_{P}}$) and liquid feed ($\dot{m_{L}}$) as well as temperature profile of the seven extruder barrel sections ($T_{2-7}$) and the die adapter ($T_{adapter}$).

**Material**	$\dot{m_{P}}$ kg/h	$\dot{m_{L}}$ kg/h	$T_{2}$ °C	$T_{3}$ °C	$T_{4}$ °C	$T_{5}$ °C	$T_{6}$ °C	$T_{7}$ °C	$T_{8}$ °C	$T_{adapter}$ °C
soy protein isolate	0.23	0.28	25	50	90	95	100	140	140	140
vital wheat gluten	0.55	0.45	25	50	90	95	100	160	160	160

**Figure A1:**
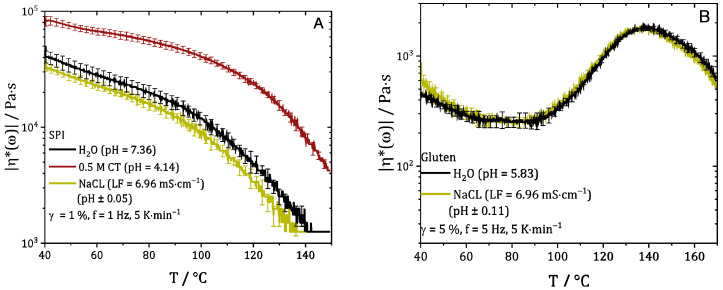
Temperature ramp of SPI (**A**) and gluten (**B**) doughs prepared with water, sodium chloride solution, and 0.5 M citric acid.

## Data Availability

The data presented in this study are openly available in article.

## References

[1] Hallström E., Röös E., Börjesson P. (2014). Sustainable meat consumption: A quantitative analysis of nutritional intake, greenhouse gas emissions and land use from a Swedish perspective. Food Policy.

[2] Apostolidis C., McLeay F. (2016). Should we stop meating like this? Reducing meat consumption through substitution. Food Policy.

[3] Kumar P., Chatli M.K., Mehta N., Singh P.r., Malav O.P., Verma A.K. (2017). Meat analogues: Health promising sustainable meat substitutes. Crit. Rev. Food Sci. Nutr..

[4] Arêas J.A. (1992). Extrusion of food proteins. Crit. Rev. Food Sci. Nutr..

[5] Cheftel J.C., Kitagawa M., Quéguiner C. (1992). New protein texturization processes by extrusion cooking at high moisture levels. Food Rev. Int..

[6] Beniwal A.S., Singh J., Kaur L., Hardacre A., Singh H. (2021). Meat analogs: Protein restructuring during thermomechanical processing. Compr. Rev. Food Sci. Food Saf..

[7] Akdogan H. (1999). High moisture food extrusion. Int. J. Food Sci. Technol..

[8] Tolstoguzov V.B. (1993). Thermoplastic extrusion-the mechanism of the formation of extrudate structure and properties. J. Am. Oil Chem. Soc..

[9] Wittek P., Ellwanger F., Karbstein H.P., Emin M.A. (2021). Morphology Development and Flow Characteristics during High Moisture Extrusion of a Plant-Based Meat Analogue. Foods.

[10] Wittek P., Zeiler N., Karbstein H.P., Emin M.A. (2021). High Moisture Extrusion of Soy Protein: Investigations on the Formation of Anisotropic Product Structure. Foods.

[11] van der Sman R.G.M., van der Goot A.J. (2023). Hypotheses concerning structuring of extruded meat analogs. Curr. Res. Food Sci..

[12] Rubinstein M., Colby R.H. (2014). Polymer Physics.

[13] Totosaus A., Montejano J.G., Salazar J.A., Guerrero I. (2002). A review of physical and chemical protein-gel induction. Int. J. Food Sci. Technol..

[14] Nisov A., Nikinmaa M., Nordlund E., Sozer N. (2022). Effect of pH and temperature on fibrous structure formation of plant proteins during high-moisture extrusion processing. Food Res. Int..

[15] Muhialdin B.J., Ubbink J. (2023). Effects of pH and aging on the texture and physicochemical properties of extruded pea protein isolate. Food Hydrocoll..

[16] Dickinson E. (2012). Emulsion gels: The structuring of soft solids with protein-stabilized oil droplets. Food Hydrocoll..

[17] Kinsella J.E., Melachouris N. (1976). Functional properties of proteins in foods: A survey. Crit. Rev. Food Sci. Nutr..

[18] Foo S.K. (2004). Effect of Temperature and pH on the Solubility of Soy Protein. Bachelor’s Thesis.

[19] Takeda K., Matsumura Y., Shimizu M. (2001). Emulsifying and Surface Properties of Wheat Gluten under Acidic Conditions. J. Food Sci..

[20] Renkema J.M.S. (2004). Relations between rheological properties and network structure of soy protein gels. Food Hydrocoll..

[21] Chen F.L., Wei Y.M., Zhang B. (2011). Chemical cross-linking and molecular aggregation of soybean protein during extrusion cooking at low and high moisture content. LWT.

[22] Gennadios A., Brandenburg A.H., Weller C.L., Testin R.F. (1993). Effect of pH on properties of wheat gluten and soy protein isolate films. J. Agric. Food Chem..

[23] Malhotra A., Coupland J.N. (2004). The effect of surfactants on the solubility, zeta potential, and viscosity of soy protein isolates. Food Hydrocoll..

[24] O’Flynn T.D., Hogan S.A., Daly D.F.M., O’Mahony J.A., McCarthy N.A. (2021). Rheological and Solubility Properties of Soy Protein Isolate. Molecules.

[25] Fischer T. (2004). Effect of extrusion cooking on protein modification in wheat flour. Eur. Food Res. Technol..

[26] Emin M.A., Quevedo M., Wilhelm M., Karbstein H.P. (2017). Analysis of the reaction behavior of highly concentrated plant proteins in extrusion-like conditions. Innov. Food Sci. Emerg. Technol..

[27] Pietsch V.L., Emin M.A., Schuchmann H.P. (2017). Process conditions influencing wheat gluten polymerization during high moisture extrusion of meat analog products. J. Food Eng..

[28] Lagrain B., Goderis B., Brijs K., Delcour J.A. (2010). Molecular basis of processing wheat gluten toward biobased materials. Biomacromolecules.

[29] Wu Y.V., Dimler R.J. (1963). Hydrogen-ion Equilibria of Wheat Gluten. Arch. Biochem. Biophys..

[30] Wittek P., Walther G., Karbstein H.P., Emin M.A. (2021). Comparison of the Rheological Properties of Plant Proteins from Various Sources for Extrusion Applications. Foods.

[31] Opaluwa C., Deskovski S., Karbstein H.P., Emin M.A. (2024). Effect of oil on the rheological properties and reaction behavior of highly concentrated wheat gluten under conditions relevant to high moisture extrusion. Future Foods.

[32] Wittek P., Zeiler N., Karbstein H.P., Emin M.A. (2020). Analysis of the complex rheological properties of highly concentrated proteins with a closed cavity rheometer. Appl. Rheol..

[33] Kendler C., Duchardt A., Karbstein H.P., Emin M.A. (2021). Effect of Oil Content and Oil Addition Point on the Extrusion Processing of Wheat Gluten-Based Meat Analogues. Foods.

[34] Langstraat T.D., Jansens K.J.A., Delcour J.A., van Puyvelde P., Goderis B. (2015). Controlling wheat gluten cross-linking for high temperature processing. Ind. Crops Prod..

[35] Shen Z., Liu Z., Rui X., Chen X., Jiang M., Dong M. (2021). Effects of fat content on the textural and in vivo buccal breakdown properties of soy yogurt. J. Texture Stud..

[36] Treloar L.R.G. (2009). The Physics of Rubber Elasticity.

[37] Saavedra Isusi G.I., Pietsch V., Beutler P., Hoehne S., Leister N. (2023). Influence of Rapeseed Oil on Extruded Plant-Based Meat Analogues: Assessing Mechanical and Rheological Properties. Processes.

[38] Meißner M. (2016). Standard-Methoden für Getreide, Mehl und Brot.

[39] Schreuders F.K.G., Sagis L.M.C., Bodnár I., Erni P., Boom R.M., van der Goot A.-J. (2021). Mapping the texture of plant protein blends for meat analogues. Food Hydrocoll..

[40] Nieuwland M., Heijnis W., van der Goot A.-J., Hamoen R. (2023). XRT for visualizing microstructure of extruded meat replacers. Curr. Res. Food Sci..

[41] Willats W.G., Orfila C., Limberg G., Buchholt H.C., van Alebeek G.J., Voragen A.G., Marcus S.E., Christensen T.M., Mikkelsen J.D., Murray B.S. (2001). Modulation of the degree and pattern of methyl-esterification of pectic homogalacturonan in plant cell walls. Implications for pectin methyl esterase action, matrix properties, and cell adhesion. J. Biol. Chem..

[42] Fraeye I., Colle I., Vandevenne E., Duvetter T., van Buggenhout S., Moldenaers P., van Loey A., Hendrickx M. (2010). Influence of pectin structure on texture of pectin–calcium gels. Innov. Food Sci. Emerg. Technol..

